# Correlation of Different Non-Invasive Neuromonitoring Tools Assessing Intracranial Hemodynamics

**DOI:** 10.3390/brainsci15070710

**Published:** 2025-06-30

**Authors:** Rossella Zangari, Luca D’Amelio, Elisa Gouvea Bogossian, Fabio Silvio Taccone

**Affiliations:** 1 Department of Intensive Care and Emergency, Spedali Civili di Brescia, Università degli Studi di Brescia, 25121 Brescia, Italy; 2 Department of Intensive Care, Route de Lennik, Erasme Hospital, Université Libre de Bruxelles, 808, 1070 Brussels, Belgium; luca.damelio91@gmail.com (L.D.); elisagobog@gmail.com (E.G.B.); fabio.taccone@ulb.be (F.S.T.)

**Keywords:** acute brain injury, intracranial hypertension, cerebral blood flow, cerebral perfusion pressure, transcranial Doppler ultrasonography, neuromonitoring, critical care

## Abstract

Background: Intracranial pressure (ICP) monitoring is crucial in managing acute brain injury (ABI) to prevent secondary brain injury. While invasive techniques remain the gold standard, they can carry notable risks, such as infection and hemorrhage. Non-invasive techniques are increasingly used, but their inter-modality correlation and concordance have not been systematically evaluated. This study aimed to assess the correlation and concordance among four commonly used non-invasive neuromonitoring tools in patients with ABI undergoing invasive ICP monitoring. Methods: This was a secondary analysis of prospectively collected data from 100 adult patients admitted to the intensive care unit with traumatic brain injury (TBI), subarachnoid hemorrhage (SAH), or intracerebral hemorrhage (ICH) who underwent invasive ICP monitoring. Simultaneous assessments using optic nerve sheath diameter (ONSD), transcranial Doppler-derived pulsatility index (PI), estimated ICP (eICP), and the neurological pupil index (NPi) were performed. Correlation between modalities was assessed using Spearman’s correlation coefficient (ρ), and concordance was evaluated with Cohen’s kappa coefficient (k). Results: We found weak correlations between ONSD and PI (ρ = 0.29), ONSD and NPi (ρ = −0.33), and PI and NPi (ρ = −0.33); moderate correlations between ONSD and eICP (ρ = 0.54) and PI and eICP (ρ = 0.48); and a strong inverse correlation between eICP and NPi (ρ = −0.71; all *p* < 0.05). Concordance was generally low, with the highest agreement between PI and eICP (k = 0.69). Most other tool pairings showed poor-to-fair concordance (k ≤ 0.30). Conclusions: Non-invasive neuromonitoring tools show variable correlation and limited agreement, suggesting they are not interchangeable. Each modality captures different aspects of cerebral physiology, supporting the use of a multimodal approach to improve accuracy in ICP estimation.

## 1. Introduction

Monitoring intracranial pressure (ICP) is crucial in the management of critically ill patients with acute brain injuries (ABIs), as intracranial hypertension remains one of the most significant determinants of secondary brain injury and poor neurological outcomes [1,2]. Timely detection and treatment of elevated ICP are essential to prevent cerebral ischemia, herniation, and neuronal damage [3]. Although invasive neuromonitoring techniques, such as intraventricular catheters and intraparenchymal sensors, are considered the gold standard for measuring intracranial pressure, their use carries some risks, including infections, hemorrhage, and technical issues requiring their management by skilled and specialized teams [4,5,6]. Moreover, their availability is limited in low/middle-income countries [7]. These limitations have contributed to growing interest in non-invasive neuromonitoring techniques that aim to provide indirect estimations of ICP and insights into cerebral hemodynamics [8,9]. Such tools represent a potentially more accessible and safer alternative to invasive monitoring, particularly in resource-limited settings or in patients where invasive approaches are contraindicated. Among the most widely studied non-invasive modalities are transcranial Doppler (TCD) ultrasound, optic nerve sheath diameter (ONSD) measurement, and automated pupillometry. Notably, each of these techniques has been independently evaluated against invasive ICP monitoring in multiple studies [10,11].

The individual application of non-invasive tools is constrained by several methodological and physiological limitations that affect their diagnostic utility in isolation [8]. ONSD measurement is subject to significant operator dependency and inter-observer variability, which can compromise its reproducibility and standardization across patient populations. Additionally, it is a static measure that may be confounded by anatomical variations and local orbital pathologies unrelated to intracranial pressure (ICP). TCCD, while valuable for assessing cerebral hemodynamics, provides only an indirect estimation of ICP through derived indices such as the pulsatility index, which are influenced by multiple factors including cerebrovascular resistance, arterial CO_2_ levels, and systemic hemodynamics. Its accuracy is further limited by the need for an adequate temporal acoustic window, which may be absent in a significant proportion of patients, and by the requirement for specialized training for accurate interpretation of waveforms. Similarly, NPi, derived from automated pupillometry, may not reliably detect early or subtle elevations in ICP, as pupillary changes often occur later in the course of neurological deterioration. Moreover, NPi readings can be influenced by some pharmacological agents (e.g., barbiturates, inhaled anesthetics), ocular trauma, or pre-existing neurological conditions, thereby reducing its specificity. As with ONSD, NPi offers only episodic assessments and lacks the capability for continuous monitoring.

Despite these limitations, while comparisons of invasive ICP with non-invasive tools have highlighted notable limitations in the sensitivity and specificity of non-invasive tools when used in isolation, emerging evidence suggests that their diagnostic accuracy improves considerably when applied in a multimodal fashion [12]. This multimodal approach, which integrates several non-invasive parameters, holds the potential to enhance clinical decision-making by providing a more comprehensive assessment of cerebral physiology. The recent B-ICONIC Consensus on non-invasive neuromonitoring, developed to facilitate the care of traumatic brain injury (TBI) patients when invasive ICP monitoring is not available, has also further emphasized the importance of a multimodal approach in neurocritical care [13], suggesting that results from different non-invasive tools can serve as valuable screening tools to identify the risk of intracranial hypertension and guide the level of therapeutic intervention. However, these tools have never been systematically compared to one another in providing information on cerebral physiology.

The aim of this study was therefore to evaluate the correlation and concordance among four commonly used non-invasive neuromonitoring tools in a cohort of critically ill ABI patients.

## 2. Materials and Methods

### 2.1. Study Design

This study represents a secondary analysis of a previously published prospective observational cohort conducted in ABI patients [14]. Ethical approval for the original study was obtained from the local institutional review board, and informed consent was waived due to the observational nature of the investigation and the use of standard monitoring techniques. The Strengthening the Reporting of Observational studies in Epidemiology (STROBE) guidelines were followed [15].

### 2.2. Study Population

Adult patients (aged ≥ 18 years) admitted to the Intensive Care Unit (ICU) department of the Hôpital Universitaire de Bruxelles (HUB) over a period of 20 months (January 2017 to September 2018) with a diagnosis of acute brain injury, including moderate to severe (Glasgow coma scale < 13) TBI, subarachnoid hemorrhage (SAH), or spontaneous intracerebral hemorrhage (ICH). Patients were consecutively enrolled if they underwent ICP monitoring as part of routine clinical management for suspected or confirmed intracranial hypertension. Patients were excluded if ICP monitoring was performed for indications unrelated to intracranial hypertension, or if they had known pupillary abnormalities, such as Adie’s pupil, Argyll Robertson pupil, postoperative anatomical distortion, glaucoma, or other conditions affecting reliable pupillary evaluation (e.g., severe periorbital edema). Additional exclusion criteria included the absence of an adequate temporal bone window for transcranial Doppler (TCD) assessment and the lack of an arterial catheter for continuous blood pressure monitoring.

Invasive monitoring was performed using either an extra-ventricular drainage (EVD) catheter or an intraparenchymal fiber-optic transducer (Neurovent, Raumedic SA, Geneva, Switzerland), as determined by the treating neurosurgical and intensivist team based on clinical indication and anatomical considerations. In conjunction with invasive ICP measurement, a standardized, multimodal non-invasive neuromonitoring protocol was applied at the bedside [14].

### 2.3. Non-Invasive ICP Assessment

The following parameters were assessed: *ONSD* was measured by ocular ultrasonography using a high-frequency linear probe placed over the closed upper eyelid, with measurements acquired 3 mm behind the retina in both eyes. The final ONSD value was calculated by averaging four measured values and abnormal ONSD was considered if ≥6.0 mm. Transcranial Doppler, using the temporal window on both sides and an echo-color Doppler device with a 2 MHz transducer; the TCD measurements were performed bilaterally on the middle cerebral artery (MCA) to calculate the Pulsatility Index (PI) and to derive estimated ICP (eICP), using a validated formula [16]. PI was considered abnormal if >1.2; eICP was considered elevated if >20 mmHg. Neurological Pupil Index (NPi) was recorded bilaterally using an automated quantitative pupillometer (NeurOptics^®^, Irvine, CA, USA), under standardized lighting and measurement conditions. Using an integrated algorithm, the pupillometer device provides the NPi, which ranges from a value of 0 to 5; an NPi score < 3 indicates abnormal pupillary function whereas NPi scores ≥ 3 are considered within the normal range.

Within the first 72 h after ICP insertion, all assessments were conducted by trained intensivists or neurocritical care practitioners with certified experience in ultrasound and pupillometry. To minimize physiological variability, non-invasive measurements were obtained during a stable clinical period and within a narrow temporal window, e.g., patients were required to have a stable ICP value, defined as <10% variation over a minimum of 30 min, and not to require any ICP-directed interventions, such as therapeutic measures, endotracheal suctioning, or other physical manipulations during this period. Concomitant therapies remained unchanged throughout the measurement interval and these data were collected when ICP did not vary by more than 3 mmHg during the measurements (around 8 min). The decision to select a period of ICP stability was based on the rationale that it would enhance the reliability and stability of measurements in the context of a study aimed at comparing different non-invasive monitoring tools.

### 2.4. Study Outcome

The primary outcome of this study was to assess the correlation and concordance among the four non-invasive neuromonitoring tools.

### 2.5. Statistical Analysis

Data are expressed as median (interquartile range) or count (percentage), as appropriate. For continuous variables, normality assumption checking was performed by inspection of residual and normal plots. Correlation between continuous non-invasive variables was assessed using Spearman’s rank correlation coefficient (ρ). The ρ was considered as “strong” (i.e., ≥0.7), “moderate” (i.e., 0.50–0.69), “weak” (0.25–0.50), or “poor” (i.e., <0.25).

Concordance between dichotomized variables (normal vs. abnormal) was evaluated using Cohen’s kappa coefficient (κ). Kappa results were interpreted as follows: values ≤ 0 as indicating no agreement, 0.01–0.20 as none to slight, 0.21–0.40 as fair, 0.41–0.60 as moderate, 0.61–0.80 as substantial, and 0.81–1.00 as almost perfect agreement [17]. Statistical significance was defined as a *p*-value < 0.05.

## 3. Results

### Study Population

A total of 195 patients underwent invasive ICP monitoring during the study period; 20 patients were younger than 18 years and 35 were excluded because ICP monitoring was inserted for ischemic stroke (*n*  =  7), hydrocephalus (*n*  =  6), or monitoring after surgery for brain tumors (*n*  =  11) or infection of ventriculo-peritoneal shunt (*n*  =  11). Of 140 eligible patients, we excluded 40 patients (unstable ICP, *n*  =  16; absent temporal window for TCD, *n*  =  15; ocular trauma, *n*  =  5; multiple sclerosis, *n*  =  1; absence of arterial catheter, *n*  =  3). Thus, 100 patients were included in the analysis, with TBI (*n*  =  30; 25 with severe TBI and 5 with moderate TBI on admission and subsequent neuroworsening), SAH (*n*  =  47), or ICH (*n*  =  23). The median age was 52 (44–62) years and 55 (55%) were male. The mean Glasgow outcome scale scores on admission and the day from ICU admission to multimodal monitoring assessment were 8 (5–12) and 7 (3–10), respectively. The characteristics of the studied population have been previously published [14] and main characteristics have been reported in Supplementary Table S1.

Median values from the different non-invasive techniques were ONSD 5.2 [4.8–5.8] mm, PI 1.1 [0.9–1.4], eICP 21 [14–29] mmHg, and NPi 4.2 [3.8–4.6]; 37 patients had ICP above 20 mmHg at the moment of the different measurements.

**Figure 1:**
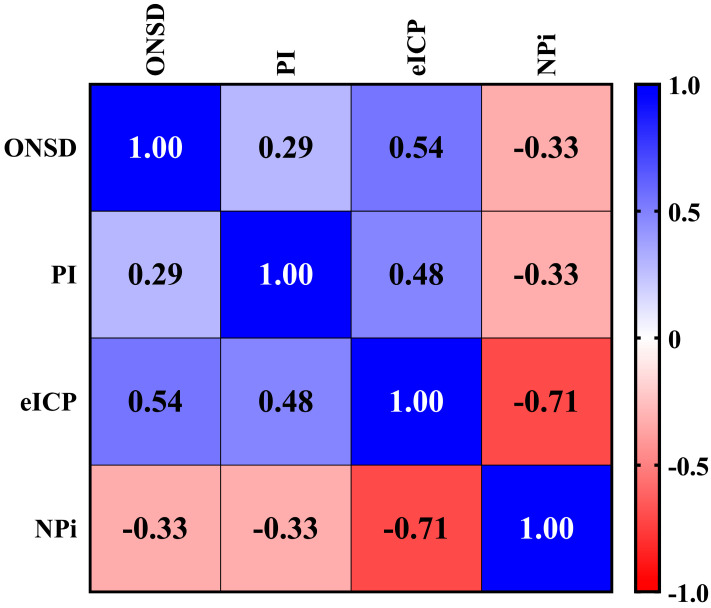
Correlation among different non-invasive neuromonitoring tools. This matrix correlation shows the different correlations among non-invasive tools commonly used to assess intracranial pressure and hemodynamics. The correlation between continuous non-invasive variables was assessed using Spearman’s rank correlation coefficient (ρ), which ranges in value from −1 (perfect negative linear relationship) to +1 (perfect positive linear relationship). The ρ was considered as “strong” (i.e., ≥0.7), “moderate” (i.e., 0.50–0.69), “weak” (0.25–0.50), or “poor” (i.e., <0.25). ONSD: Optic Nerve Sheath Diameter. PI: Pulsatility Index. eICP: estimated Intracranial Pressure. NPi: Neurological Pupil index.

We found a statistically significant and strong inverse correlation between eICP and NPi (ρ = −0.71; *p* < 0.05), as shown in Figure 1. ONSD (ρ = 0.54; *p* < 0.05) and PI (ρ = 0.48; *p* < 0.05) were moderately directly correlated with eICP. A weak correlation was found between ONSD and PI (ρ = 0.29, *p* < 0.05), ONSD and NPi (ρ = −0.33, *p* < 0.05), and PI and NPi (ρ = −0.33, *p* < 0.05).

Table 1 reports the concordance among different non-invasive neuromonitoring methods. We observed a poor concordance between ONSD and eICP (k = 0.20); a fair concordance between ONSD and PI (k = 0.27), eICP and NPi (k = 0.30), or between ONSD and NPi (k = 0.29); a moderate concordance between PI and NPi (k = 0.46); a good concordance between PI and eICP (k = 0.69).

## 4. Discussion

In this study, we observed a variable and generally weak-to-moderate correlation, along with limited concordance reflecting different physiological dimensions, among several commonly used non-invasive neuromonitoring tools employed to estimate ICP. These findings underscore the fact that these modalities are not interchangeable and should be considered complementary rather than alternative methods. A multimodal approach that integrates multiple tools may improve the accuracy of ICP estimation and enhance clinical decision-making in neurocritical care settings.

The observed weak correlation and concordance are likely attributable to the fact that each non-invasive tool assesses distinct physiological dimensions of intracranial dynamics. For example, ONSD measurement primarily reflects cerebrospinal fluid (CSF) pressure dynamics and potential obstruction of CSF outflow [18,19]. In contrast, TCD ultrasound evaluates cerebral blood flow velocities, serving as a surrogate for global intracranial hemodynamics, which is influenced by either systemic (e.g., hypotension) and/or cerebral mechanisms (e.g., increased ICP, hyperventilation) [20,21]. Automated pupillometry, on the other hand, provides an objective assessment of brainstem function by quantifying pupillary light reflexes, which can be sensitive to rising ICP and impending herniation [22,23]. Each of these modalities therefore contributes a unique and complementary perspective on the complex pathophysiological processes underlying elevated ICP and secondary cerebral injury. These differences highlight the importance of adopting a multimodal neuromonitoring strategy, which leverages the strengths of individual tools while compensating for their limitations, rather than relying on any single parameter in isolation.

Very few studies have systematically assessed the correlation and concordance among non-invasive neuromonitoring tools in critically ill patients. In a pilot study of tuberculous meningitis, an inverse relationship was found between ONSD and NPi measurements, both of which were associated with initial disease severity and predictors of poor outcome [24]. The B-ICONIC Consensus on non-invasive neuromonitoring [13] underscored the necessity of a multimodal approach to enhance the accuracy and reliability of ICP estimation in the absence of invasive neuromonitoring. In this evolving landscape, our study, which systematically evaluates the correlation and concordance among different non-invasive neuromonitoring tools, is particularly timely and well-positioned. By directly addressing one of the key gaps identified in the B-ICONIC Consensus, e.g., the lack of comparative studies between these modalities, our findings contribute to the ongoing efforts to refine multimodal non-invasive monitoring strategies. Future studies should focus on validating this multimodal approach and exploring its impact on patient outcomes in a variety of neurocritical care settings.

This study has several limitations that should be acknowledged. First, as a secondary analysis, we were restricted to the dataset collected in the original study. As such, we were unable to fully account for several potential confounding factors, including arterial carbon dioxide tension, neuroimaging findings, body temperature, and the cumulative doses of sedatives or other medications, all of which may significantly influence ICP and cerebral physiology.

Second, there was no standardized timing for the neuromonitoring measurements across patients. The timing of assessments was variable and based on clinical circumstances, which may have introduced bias or variability into our findings. However, it is important to note that, for each individual patient, all non-invasive and invasive monitoring techniques were performed concurrently, thereby reducing intra-patient temporal variability.

Third, the majority of measurements were obtained in patients with well-controlled ICP, which limits the generalizability of our results to patients experiencing dynamic or poorly controlled intracranial hypertension. Consequently, our findings should not be extrapolated to scenarios involving significant ICP fluctuations without further validation.

Additionally, we did not examine temporal trends in ICP or assess how changes over time were reflected by the various non-invasive neuromonitoring methods. Evaluating the sensitivity of these tools to detect clinically relevant changes in ICP over time would provide important insights but was beyond the scope of the current study.

The total duration of ICP monitoring is a relevant factor when interpreting the strength and nature of correlations observed between invasive and non-invasive parameters. In our study, non-invasive assessments were performed during predefined periods of clinical and ICP stability, rather than across the entire duration of invasive monitoring and this methodological choice was made to reduce variability and enhance measurement reliability in the context of a comparative tool validation study. While prolonged ICP monitoring can provide valuable insights into dynamic intracranial physiology, including transient fluctuations or delayed trends, our design focused on controlled, stable windows to allow for more consistent comparison across modalities. We acknowledge, however, that the overall duration of invasive monitoring may influence the generalizability of our findings to more variable clinical scenarios. Future studies incorporating time-series analyses over extended monitoring periods may offer a more nuanced understanding of how non-invasive indices track with ICP changes over time.

Moreover, the heterogeneity of the studied population may have impacted our results. Due to the small sample size, we did not perform subgroup analysis regarding stratified by patients’ characteristics such as brain injury etiology, sex, and Glasgow coma scale. Similarly, we did not assess the impact of the length of time between admission and start of invasive monitoring, admission and acquisition of non-invasive measurements, and between surgery/intervention timing and start of invasive monitoring.

Finally, for measurements such as ONSD or NPi, we used averaged values from both eyes, a common approach in the literature. However, this method may be suboptimal, as side-specific measurements—such as those taken from the side of the primary brain injury or the side where the invasive ICP probe was placed—might yield more accurate correlations. Future studies should consider evaluating the diagnostic utility of lateralized ONSD measurements in relation to focal pathology.

## 5. Conclusions

In this study, non-invasive neuromonitoring tools commonly used to estimate ICP demonstrated variable correlation and limited concordance with one another, indicating that they are not interchangeable. Each modality reflects distinct aspects of cerebral physiology—such as cerebrospinal fluid dynamics (ONSD), cerebral vascular resistance (PI), or brainstem function (NPi)—underscoring their complementary rather than redundant roles.

These findings support the implementation of a multimodal monitoring strategy in clinical practice, particularly in settings where invasive ICP monitoring is not available or is contraindicated. Rather than relying on a single non-invasive parameter, integrating multiple tools may enhance diagnostic confidence and help guide clinical decision-making, because of their complementarity. Clinicians should be aware of the physiological scope and limitations of each modality, and use them contextually to support a more comprehensive assessment of intracranial dynamics. Further research is warranted to define condition-specific thresholds and to develop standardized protocols that incorporate these tools into routine neurocritical care algorithms.

## Figures and Tables

**Table 1:** Concordance among different non-invasive neuromonitoring tools. This table shows the different concordances among non-invasive tools commonly used to assess intracranial pressure. The concordance was described using Cohen’s coefficient (κ), which is often interpreted as follow: values ≤ 0 as indicating no agreement, 0.01–0.20 as none to slight, 0.21–0.40 as fair, 0.41–0.60 as moderate, 0.61–0.80 as substantial, and 0.81–1.00 as almost perfect agreement. ONSD: Optic Nerve Sheath Diameter. PI: Pulsatility Index. eICP: estimated Intracranial Pressure. NPi: Neurological Pupil index.

Variables	Cohen’s Kappa Coefficient
ONSD vs. eICP	0.20
ONSD vs. PI	0.27
ONSD vs. NPi	0.29
eICP vs. NPi	0.30
PI vs. NPi	0.46
eICP vs. PI	0.69

## Data Availability

The original contributions presented in this study are included in the article and Supplementary Material (patients characteristics, statistical analysis). Further inquiries can be directed to the corresponding author.

## Supplementary Materials

The following supporting information can be downloaded at: https://www.mdpi.com/article/10.3390/brainsci15070710/s1, Table S1: Characteristics of the studied population.

## Abbreviations

The following abbreviations are used in this manuscript:

ABI	Acute Brain Injury
EVD	External Ventricular Drain
eICP	Estimated Intracranial Pressure
ICH	Intracerebral Hemorrhage
ICP	Intracranial Pressure
NPi	Neurological Pupil index
ONSD	Optic Nerve Sheath Diameter
PI	Pulsatility Index
SAH	Subarachnoid Hemorrhage
TBI	Traumatic Brain Injury
TCCD	Transcranial Color Doppler
ICU	Intensive Care Unit
