# Supplementary material for: Correlation of Different Non-Invasive Neuromonitoring Tools Assessing Intracranial Hemodynamics
Source: Brain Sci. 2025 Jun 30;15(7):710. doi: 10.3390/brainsci15070710 (PMC12293322; doi:10.3390/brainsci15070710)
Supplement: Supplementary file 1 [file brainsci-15-00710-s001.zip › brainsci-3696084-supplementary.pdf]

Supplementary Table S1. Characteristics of the patients included in our cohort, according to underlying brain diseases.

	**ALL (n=100)**
**Age, years**	52 [44-62]
**Male, n (%)**	55 (55)
**ICU stay, days**	16 [11-23]
**GCS on admission**	8 [5-12]
**Days from admission to ICP assessment**	2 [2-3]
**COMORBIDITIES**	
Chronic heart failure, n (%)	2 (2)
Diabetes, n (%)	8 (8)
COPD/asthma, n (%)	6 (6)
Liver cirrhosis, n (%)	4 (10)
HIV, n (%)	2 (2)
Cancer, n (%)	1 (1)
Alcohol, n (%)	16 (16)
**ON ADMISSION**	
**Brain Injury**	
Traumatic Brain Injury, n (%)	30 (30)
Subarachnoid Hemorrhage, n (%)	47 (47)
Intracranial Hemorrhage, n (%)	23 (23)
**Sedatives, n (%)**	39 (39)
**Opioids, n (%)**	55 (55)
**Barbiturates, n (%)**	7 (7)
**Vasopressors, n (%)**	60 (60)
**NMBAs, n (%)**	11 (11)
**ON THE DAY OF ASSESSMENT**	
**Intracranial pressure, mmHg**	17 [12-25]
**Intracranial hypertension, n (%)**	37 (37)
**Mean ONSD, mm**	5.2 [4.8-5.8]
**Mean CBFDV, cm/sec**	44 [34-56]
**Mean PI**	0.93 [0.83-1.16]
**eICP, mmHg**	18 [13-24]
**Mean NPI**	4.2 [3.8-4.6]
**OUTCOMES**	
**ICU mortality, n (%)**	30 (30)

ICU = Intensive Care Unit; GCS = Glasgow Coma Scale; COPD = chronic obstructive pulmonary disease; HIV = human immunodeficiency virus; NMBA = neuromuscular blocking agents; ONSD = optic nerve sheath diameter; CBFDV = cerebral blood flow diastolic velocity; PI = pulsatility index; ICP = intracranial pressure; eICP = estimated intracranial pressure; NPI = neurological pupil index; MV = mechanical ventilation; GOS = Glasgow Outcome Scale
